# Indolent B-Cell Lymphomas Associated with HCV Infection: Clinical and Virological Features and Role of Antiviral Therapy

**DOI:** 10.1155/2012/638185

**Published:** 2012-08-26

**Authors:** Luca Arcaini, Michele Merli, Stefano Volpetti, Sara Rattotti, Manuel Gotti, Francesco Zaja

**Affiliations:** ^1^Department of Hematology Oncology, University of Pavia, Fondazione IRCCS Policlinico San Matteo, 27100 Pavia, Italy; ^2^Division of Hematology, Department of Internal Medicine, Ospedale di Circolo, Fondazione Macchi, 21100 Varese, Italy; ^3^Department of Hematology, DISM, Azienda Ospedaliero Universitaria S. M. Misericordia, 33100 Udine, Italy; ^4^Clinica Ematologica, Centro Trapianti e Terapie Cellulari “Carlo Melzi”, DISM, Azienda Ospedaliero Universitaria S. M. Misericordia, p.le S. Maria Misericordia 15, 33100 Udine, Italy

## Abstract

The association between hepatitis C virus (HCV) infection and B-cell non-Hodgkin's lymphomas (NHL) has been demonstrated by epidemiological studies, in particular in highly endemic geographical areas such as Italy, Japan, and southern parts of United States. In these countries, together with diffuse large B-cell lymphomas, marginal zone lymphomas are the histotypes most frequently associated with HCV infection; in Italy around 20–30% cases of marginal zone lymphomas are HCV positive. Recently, antiviral treatment with interferon with or without ribavirin has been proved to be effective in the treatment of HCV-positive patients affected by indolent lymphoma, prevalently of marginal zone origin. An increasing number of experiences confirmed the validity of this approach in marginal zone lymphomas and in other indolent NHL subtypes like lymphoplasmacytic lymphoma. Across different studies, overall response rate was approximately 75%. Hematological responses resulted significantly associated with the eradication of the virus. This is the strongest evidence of a causative link between HCV and lymphomas. The aim of this paper is to illustrate the relationship between HCV infection and different subtypes of indolent B-cell lymphomas and to systematically summarize the data from the therapeutic studies that reported the use of antiviral treatment as hematological therapy in patients with HCV-associated indolent lymphomas.

## 1. Introduction

In the last two decades, evidences from either epidemiological studies, biological insights, and also therapeutic approaches provided strong support to the association between hepatitis C virus (HCV) and B-cell non-Hodgkin's lymphomas (NHL). HCV has been associated with B-cell indolent lymphomas, especially marginal zone lymphomas, as well as with aggressive lymphomas, mainly diffuse large B-cell lymphomas. Indolent lymphomas are defined from a clinical point of view as scarcely symptomatic lymphomas, growing and spreading slowly [1] and encompass the following histologic subtypes of low-grade lymphoma according to the WHO classification [2]: follicular lymphoma, small lymphocytic lymphoma, marginal zone lymphomas, splenic marginal zone lymphoma, primary nodal marginal zone lymphoma and extranodal marginal zone lymphoma of mucosa-associated tissue (MALT), and lymphoplasmacytic lymphoma. According to the currently more accepted pathogenetic model, the role of HCV infection in lymphomagenesis may be related to the chronic antigenic stimulation of B-cell immunologic response by the virus [3], similarly to the well-characterized induction of gastric MALT lymphoma development by *Helicobacter pylori* chronic infection [4]. In a similar way, chronic HCV infection may possibly sustain a multistep evolution from type II mixed cryoglobulinemia to overt low-grade NHL and eventually to high-grade NHL [3, 4]. The most convincing argument in favour of a causative link between HCV and lymphoproliferation is represented by interventional studies demonstrating that in HCV-positive patients affected by indolent NHL eradication of HCV with antiviral treatment (AT) could directly induce lymphoma regression [5]. Moreover, the upcoming novel antiviral anti-HCV agents as boceprevir and telaprevir, whose addition to standard treatment has already demonstrated an increased rate of viral eradication also in more resistant genotypes (i.e., genotype 1b) [6, 7], will possibly further improve the efficacy of this treatment for HCV-positive indolent NHL in the near future. 

## 2. Methods

The aim of this paper is to systematically summarize the available data indolent B-cell NHL associated with HCV infection and the up-to-now reported experiences with the use of AT with interferon with or without ribavirin as hematologic treatment in patients with HCV-positive indolent B-cell NHL.

 To this aim, we performed a systematic PubMed search (http://www.pubmed.gov/) using the keywords “indolent lymphoma,” “marginal zone lymphoma,” “MALT lymphoma,” “lymphoplasmacytic lymphoma,” “hepatitis C virus,” “interferon,” “ribavirin,” “antiviral therapy.” All relevant articles were included, as well as the most significant abstracts presented at American Society of Hematology (ASH) meetings and International Conference on Malignant Lymphomas (ICML) meetings since 2005. The articles were reviewed with reference to the features of HCV-associated indolent NHL and were assessed specifically concerning virological and hematological response in cases treated with AT.

## 3. HCV Infection, Cryoglobulinemia, and ****Lymphomas

### 3.1. HCV and Cryoglobulinemia

The initial finding that lead to the extensive investigation of the association between HCV and NHL was the very high prevalence (nearly 90–100%) of HCV infection in patients with type II mixed cryoglobulinemia [8]. Cryoglobulins are serum immunoglobulins that become insoluble and precipitate at temperatures below 37°C. The antigenic component of the immune complexes has been found to be highly enriched in viral HCV core protein and HCV-RNA. Type II mixed cryoglobulinemia is characterized by a mixture of monoclonal and polyclonal immunoglobulins. The monoclonal component of type II mixed cryoglobulinemia is an IgM/k with a rheumatoid-factor activity (i.e., anti-IgG cross-reactive binding) that reflects the expansion of a B-cell monoclonal population [9]. Overall, up to 50% of HCV-infected patients exhibit low levels of circulating mixed cryoglobulins, whereas overt cryoglobulinemic vasculitis develops in ≤5% of infected patients [10]. Symptoms vary from purpura and arthralgia to more severe manifestations like peripheral neuropathy and glomerulonephritis. Importantly, in HCV-infected patients with symptomatic cryoglobulinemia, the risk to develop an NHL is greatly increased with respect to the general population (about 35 times according to a multicenter Italian study) [11]. As a result, approximately 8–10% of patients with type II mixed cryoglobulinemia ultimately progress to a frank NHL after long-term followup. Type II mixed cryoglobulinemia is often characterized by IgH or Bcl2 rearrangement and by t(14; 18) translocation [12]. Treatment of HCV type II mixed cryoglobulinemia with severe organ damage may target either the viral trigger (HCV) or the downstream arm of B-cell autoimmunity. AT with pegylated interferon + ribavirin has been shown to reverse bone marrow B-cell expansion in patients with HCV-MC, leading to a clinical and virologic response in up to 60% of cases [13]. Recent studies showed that the combination of AT (PEG-interferon + ribavirin) with the anti-CD20 monoclonal antibody rituximab is well tolerated and more effective than AT alone, by increasing the rate of complete clinical response, immunologic (cryoglobulin clearance) and molecular response (eradication of B-cell clonal expansions), and shortening the time to achieve a complete clinical response [14, 15].

### 3.2. Epidemiology of HCV and Its Association with Lymphomas 

HCV infection is a global health problem, with up to 170 million persons infected worldwide (3% of global population) [25]. However relevant regional differences in the prevalence of infection are described. The lowest prevalence rates are reported in Northern Europe and Scandinavia (0.01–0.1%) while in Italy, Egypt, Japan, and southern parts of United States, prevalence estimates exceeds 2% [26]. HCV infection is a leading cause of chronic hepatitis, cirrhosis, and hepatocellular carcinoma and has been associated to extrahepatic manifestations, especially type II mixed cryoglobulinemia and a spectrum of B-cell NHL with or without cryoglobulinemia [3]. Several epidemiological studies have been performed beginning from the mid 1990s to investigate the link between HCV and NHL. Early studies based on relatively small number of cases suggested a significant increased risk of B-cell NHL in HCV-positive patients, especially in countries with high prevalence of HCV infection as Italy [27], Japan, and southern regions of USA, while studies from areas with low HCV prevalence did not show any evident association [28]. In 2003, a systematic review of studies evaluating prevalence of HCV infection in B-cell NHL [29] was published. In this report, 48 studies (5,542 patients) were identified. Mean HCV infection prevalence was 13%. In 10 case-control studies examined, HCV prevalence in B-cell NHL was 17% compared to 1.5% in healthy controls (odds ratio, OR = 10.8). Therefore, it was concluded that HCV prevalence in patients with B-cell NHL is higher than that reported in general population (15% vs. 1.5%), suggesting a role of HCV in the aetiology of B-cell NHL. Subsequently, in 2006, an updated metaanalysis of 15 case-control studies on the association between HCV infection and NHL demonstrated a pooled relative risk of lymphoma among HCV-positive subjects of 2–2.5 depending on study design. Relative risks resulted consistently increased for all major B-NHL subtypes. The study, indeed, confirmed that the risk to develop NHL in the context of HCV infection is most dramatically evident in populations with high HCV prevalence and that consequently the fraction of NHL attributable to HCV infection varies greatly by country, reaching 10% in highly endemic areas. 

 The numbers of cases analyzed in these series were too small to establish a correlation between HCV and specific histotypes. In the Epilymph [30] case-control study, the subtype most clearly associated with HCV infection was diffuse large B-cell lymphoma, followed by marginal zone lymphoma and lymphoplasmacytic lymphoma; however these results were based on relatively few cases. To obtain a more robust estimate of the risk to develop specific NHL subtypes after HCV infection, the International Lymphoma Epidemiology Consortium (InterLymph), based in Europe, North America, and Australia, performed a pooled case-control study including in the analysis data of 7 previous surveys [31]. Overall, among 4,784 cases of NHL and 6,269 controls matched by sex, age, and study centre, HCV infection was detected in 172 NHL cases (3.6%) and in 169 (2.7%) controls. In subtype-specific analyses, HCV prevalence was associated with diffuse large B-cell lymphoma (OR 2.24), marginal zone lymphoma (OR, 2.47), and lymphoplasmacytic lymphoma (OR 2.57) whereas risk estimates were not increased for follicular lymphomas (OR 1.02). Moreover, also retrospective series reported a high HCV prevalence among patients with marginal zone lymphoma [4, 16–18, 20, 32], lymphoplasmacytic lymphoma, and diffuse large B-cell lymphoma [33, 34].

## 4. HCV and Indolent Lymphomas: Clinical and**** Pathological Studies

As mentioned above, many well-characterized clinical-pathological studies investigated the association of HCV infection with specific indolent NHL subtypes (Table 1).

### 4.1. Marginal Zone Lymphomas

Within specific indolent NHL subtypes, the association with HCV infection has been best characterized in marginal zone lymphomas. In the 2008 edition of WHO classification three marginal zone lymphoma entities were listed [2]: splenic B-cell marginal zone lymphoma, nodal marginal zone lymphoma, and extranodal marginal zone B-cell lymphoma of MALT type. Marginal zone B-cells have been demonstrated to play role in the immune response to T-cell-independent antigens and frequently display reactivity to self antigens. Marginal zone B-cells are involved in various infectious and autoimmune conditions and marginal zone-related neoplasms often retain the features of these cells. Many infectious agents are involved in the pathogenesis of specific types of marginal zone lymphomas: *Helicobacter pylori* for gastric MALT lymphoma [4], *Campylobacter jejuni* for immunoproliferative small intestine disease [35], *Borrelia burgdorferi* for MALT lymphoma of the skin [36], and *Chlamydia psittaci* for MALT lymphoma of the orbit [37, 38]. In all these cases, eradication of the antigen after antimicrobial therapy may lead to a regression of the lymphoma. For example, eradication of *Helicobacter pylori* leads to the complete regression of gastric MALT lymphoma in most cases, and relapse of the lymphoma is anticipated by the reoccurrence of the infection [4]. 

 Accordingly to this scenario, also chronic stimulation by HCV may play a role in development of a subgroup of marginal zone lymphoma cases. The association between HCV and marginal zone lymphomas is demonstrated by epidemiological studies, as previously summarized, and by clinical-pathological studies of well-characterized marginal zone lymphoma series. However, the major support to the potential causal role of HCV in marginal zone lymphomagenesis is represented by the antilymphoma activity of AT evidenced in a subset of HCV-positive MZL.

### 4.2. Splenic Marginal Zone Lymphoma

Splenic marginal zone lymphoma is a rare indolent lymphoma subtype which accounts for less than 2% of all NHL [39]. In some cases lymphocytes with villous projections are found in peripheral blood, and the disease is termed *splenic lymphoma with villous lymphocytes* [40, 41]. This entity is considered as the leukemic counterpart of splenic marginal zone lymphoma [42]. In almost all patients a symptomatic splenomegaly is the presenting feature. In a large series, HCV serology was positive in 49 out of 255 available patients (19%) [16]. Among 56 patients tested for HCV-RNA, 25 (45%) were positive. Cryoglobulins were detected in 13 out of 130 patients tested (10%).

 In 2005, French authors reported a series of splenic lymphomas with villous lymphocytes associated with type II cryoglobulinemia and HCV infection [17]. All 18 patients had type II mixed cryoglobulinemia, with symptoms of vasculitis in 13. Clinical symptoms of cryoglobulinemia preceded the diagnosis of lymphoma in 7 patients (at a mean time of 3.5 years before lymphoma diagnosis) and were concurrent in the other 6 patients. The authors concluded that splenic lymphoma with villous lymphocytes could be integrated in the spectrum of cryoglobulin-associated B-cell proliferations, configuring a new clinical entity. 

### 4.3. Primary Nodal Marginal Zone Lymphoma 

Primary nodal marginal zone lymphoma is listed in the WHO lymphoma classification as a rare but distinct clinical pathologic subtype characterized by exclusive primary lymph node localization in absence of prior or concurrent extranodal site of involvement. Primary nodal marginal zone lymphoma is a rare disease accounting for nearly 2% of lymphoid neoplasms and is frequently associated with HCV infection with preferential use of specific VH segments [43]. In a large series [18], HCV serology was positive in 9 out of 38 patients (24%) and HCV-RNA was detectable in 4/8 patients studied. 

### 4.4. MALT Lymphoma

MALT lymphomas represent 8% of all NHL [44–46], behave usually as indolent diseases, and develop more frequently in middle and advanced age, with a female predominance [47–50]. Interestingly, some studies reported an increased prevalence of HCV infection in unselected patients with gastric lymphoma [51]. In an Italian multicenter study, data on HCV serology were available in all 172 cases of nongastric MALT lymphoma [32]. HCV infection was documented in 60 patients (35%). A total of 22 out of 24 patients tested (92%) had viremia. Interestingly, three specific MALT lymphoma sites showed an elevated prevalence of HCV infection: salivary glands (47%), skin (43%), and orbit (36%). These data, while confirming the link between HCV infection and salivary glands lymphoma [52], reveal a possible relationship between HCV and two other MALT presentations of lymphoma: orbit and skin. Interestingly, a study on B-cell lymphoma in patients with Sjögren's syndrome and HCV infection reported an elevated occurrence of parotid involvement and a high proportion of MALT lymphomas with primary extranodal involvement (exocrine glands, liver, and stomach) [53]. In 2006, Ferreri et al. found HCV seropositivity in 13% of ocular adnexa lymphoma of MALT type with a more aggressive behaviour [20]. Taken together, these data indicate that the typical presentation of HCV-related MALT lymphomas is constituted by some well-defined forms with a single and peculiar MALT localization. At this regard, it has recently reported a series of 12 HCV-positive patients presenting with subcutaneous nodules resembling “lipomas” and a typical histology of extranodal marginal zone lymphoma of MALT [21]. HCV-RNA was detectable in all 10 patients tested. From a clinical point of view, it has to be underlined that the clinical benign appearance of these “lipoma-like” lesions and their indolent clinical behaviour may result in diagnostic delay.

### 4.5. Lymphoplasmacytic Lymphoma/Waldenstrom Macroglobulinemia

Beside marginal zone lymphomas, one of the other indolent B-cell NHL subtypes that has been frequently associated to HCV infection is lymphoplasmacytic lymphoma, a neoplasm of small B lymphocytes, plasmocytoid lymphocytes, and plasma cells, usually involving bone marrow and sometimes lymph nodes and spleen. Waldenstrom macroglobulinemia is found in a significant proportion of patients with lymphoplasmacytic lymphomas and is defined as lymphoplasmacytic lymphoma with bone marrow involvement and the detection of a paraprotein of IgM type in the serum. Lymphoplasmacytic lymphoma and Waldenstrom macroglobulinemia have been associated with HCV infection and mixed cryoglobulinemia in some but not all series, perhaps related to geographic differences. For example, while a US series did not find any HCV-positive cases among 100 untreated patients affected by Waldenstrom macroglobulinemia [54], an Italian multicentre study [22] reported 21 HCV-positive cases among 140 patients (15%). HCV infection was associated to lower counts of platelets, neutrophil granulocytes, and hemoglobin, and with the presence of cryoglobulins, splenomegaly, increased LDH, and *β*_2_-microglobulin levels. However, the analyses did not reveal any difference between HCV-positive and HCV-negative patients in terms of “disease progression needing treatment,” “time from diagnosis to first therapy,” and overall survival. Interestingly, a recent report specifically focused on the comparison between Waldenstrom macroglobulinemia and splenic marginal zone lymphoma, found that, despite some common features, splenic marginal zone lymphoma displayed a clearly higher association with HCV infection (25 HCV-positive patients out of 92, 27%) than Waldenstrom macroglobulinemia (6/66 patients, 9%) [55].

### 4.6. B-Cell Chronic Lymphoproliferative Disorders 

Follicular lymphoma and small lymphocytic lymphoma are low-grade lymphoma subtypes rarely associated with HCV-positive infection, as evidenced by the above mentioned epidemiological studies. Interestingly, these findings have been confirmed by a single institution Bayesian analysis that was performed to estimate the prevalence of HCV infection across the different NHL histologies. This approach was able to demonstrate the association of splenic marginal zone lymphoma and diffuse large B-cell lymphoma with HCV infection, while did not find any correlation with follicular lymphoma and small lymphocytic lymphoma [56], thus confirming previous findings of classic epidemiologic studies. Moreover, this study showed that another disease entity could be associated with HCV, the so-called “B-cell chronic lymphoproliferative disorders.” B-cell chronic lymphoproliferative disorders are defined as the miscellaneous category of non-CLL leukemic lymphoproliferative disorders with Royal Marsden Hospital scoring system ≤3 [57, 58] and are often reported also as “low-grade B-NHL not otherwise specified.” Although one series reported a low rate of HCV positivity (5%) in CD5/CD10-negative B-cell chronic lymphoproliferative disorders [24], further investigations are needed to elucidate this issue, given the heterogeneity and the small numbers of studies focusing on this entity now available. 

## 5. Standard Treatment for HCV Chronic**** Hepatitis

The goal of AT in HCV-related chronic hepatitis is to prevent disease complications. This is best accomplished through virus eradication, defined as sustained virologic response (SVR), that is, undetectable HCV-RNA by a sensitive polymerase-chain-reaction- (PCR-) based assay 24 weeks after discontinuation of treatment. Therapeutic options for HCV-related chronic hepatitis have improved significantly since the introduction of interferon monotherapy. The current standard of care is a combination of pegylated interferon-*α* and weight-based ribavirin for 48 weeks for genotype 1 and 4 and for 24 weeks for genotype 2 and 3. Patients with genotype 2 or 3 respond more favourably to standard AT, obtaining a SVR in 75–90% of cases, whereas in genotype 1 and 4 the likelihood of achieving SVR is considerably lower (45–52%) [59–61]. Recently, however, the introduction of the HCV NS3/4A protease inhibitors boceprevir [6] and telaprevir [7], the first two drugs belonging to a new and promising generation of direct-acting antiviral agents, has been demonstrated to dramatically improve SVR rates in genotype 1 patients. In particular, in genotype 1 treatment-naive patients, the addition of boceprevir or telaprevir to standard AT increased the rate of SVR to nearly 65–75%, whereas in relapsers or nonresponders, the SVR rate improved to nearly 57–86%. 

Other new potent protease inhibitors showing promising activity are currently in late phases of development, as well as other new upcoming classes of direct-acting antiviral agents like polymerase inhibitors: their potential combination seems to herald for the near future the possibility to obtain highly efficacious interferon-free regimens for HCV therapy [62].

## 6. Antiviral Treatment of HCV-Positive Indolent Lymphomas

As previously discussed, case-control epidemiological studies and clinical-pathological series evidenced the link between HCV infection and NHL development. However, the most convincing evidence to support the causal role of HCV in lymphomagenesis is the possible regression of indolent lymphoma after eradication of HCV infection with AT. Beginning from the seminal work by Hermine et al. in splenic lymphomas with villous lymphocytes in 2002 [5], data from literature demonstrate that AT could be considered as first-line approach in HCV-associated indolent lymphomas when there is not immediate need of conventional (immuno)-chemotherapy treatment. Among specific NHL subtypes, this treatment modality has been more frequently exploited in marginal zone lymphomas; however other studies have supported the validity of this approach for all indolent histologies when associated to HCV infection. In Table 2 we summarized results of antilymphoma activity of AT with interferon with or without ribavirin in low-grade NHL.

Conversely, front-line AT is clearly insufficient in HCV-positive aggressive lymphomas, in which an immediately active therapy is needed; in these cases AT may be a rationale recommendation after completion of conventional immunochemotherapy with the aim to clear a potential lymphoma trigger. 

### 6.1. The First Experiences

In 2005, a systematic review concerning the efficacy of AT in lymphoproliferative disorders was published [76]. Overall, 16 studies reporting the employment of an antiviral regimen (interferon with or without ribavirin) as primary hematologic treatment in 65 HCV-infected patients diagnosed with a lymphoproliferative disorder were identified. Complete remission of the lymphoproliferative disorder was achieved in 75% of the cases. However, many of these series relied on case reports of few patients and included also patients with mixed cryoglobulinemia with evidence of B-cell monoclonality [77].

 Among the studies included in the cited review, the first experience of a relatively large cohort of patients with NHL was performed by Hermine et al. in 2002 [5]. In this report they described the outcome of 9 patients with splenic lymphoma with villous lymphocytes and HCV infection treated with interferon-*α*2b (3 MU subcutaneously three times a week for six months). Six patients had symptomatic cryoglobulinemia. Complete hematological remission and HCV negativity were observed in 7/9 (78%) patients. Two patients who did not respond were subsequently treated with interferon plus ribavirin (1,000 to 1,200 mg per day) and obtained the clearance of HCV-RNA as well as lymphoma response (one complete remission and one partial remission). On the contrary, none of 6 cases with SLVL without HCV infection treated with interferon experienced any grade of lymphoma regression. 

 In 2005 a subsequent report from the same group expanded these results in 18 patients with chronic HCV infection, mixed cryoglobulinemia, and splenic lymphoma with villous lymphocytes [17]. All patients were treated with interferon (+ribavirin in 10). Fourteen patients obtained a complete hematologic remission after clearance of HCV-RNA. Two patients had a virologic partial response and obtained a complete hematologic response. Two virologic nonresponders achieved partial hematologic response. Notably, viral genotype did not seem to correlate with the response: in fact 4 out of 7 patients with HCV genotype 1, that is usually associated with poor responses, achieved a complete lymphoma regression after interferon and ribavirin. Interestingly, even for patients who exhibited a complete hematological remission, no molecular response was evidenced, as monoclonal immunoglobulin gene rearrangement was still detected in peripheral blood after treatment; however clinical relapses did not occur if viremia remained negative. Overall, these observations may suggest differences in oncogenic potential between HCV-driven B-cell clones in cryoglobulinemia with respect to splenic lymphoma and are in favour of a model in which a continuous viral stimulation leads to cryoglobulinemia and, subsequently, in a subset of patients, to indolent lymphoma.

 Another study reported first-line AT with interferon and ribavirin in 8 HCV-positive patients with different subtypes of marginal zone lymphoma (4 splenic marginal zone lymphomas with or without villous lymphocytes, 1 disseminated marginal zone lymphoma, 1 leukemic marginal zone lymphoma, and 2 intestinal MALT lymphomas); 5 out of 8 patients (60%) obtained a response, which was correlated to virological response in most cases [70]. 

 Among most robust experiences, an Italian multicenter study [71] reported results of AT in 13 indolent B-cell NHL (4 splenic marginal zone lymphomas, 2 primary nodal marginal zone lymphomas, 2 extranodal lymphomas of MALT, 4 lymphoplasmacytic lymphomas, and 1 follicular lymphoma) carrying HCV infection. All patients underwent AT alone with PEG-interferon and ribavirin, 10 as first line and 3 as second or third line of therapy. Eleven patients completed planned treatment, while 2 discontinued it because of severe adverse effects. Among 12 assessable patients, 7 achieved complete response, 2 partial response (overall response rate 75%), 2 had stable disease, and one progressed during therapy. Hematologic responses resulted highly significantly associated to clearance or decrease in serum HCV viral load, as 7 out of 9 achieved SVR, one had reduction in viremia, and 1 had no change in viral load. Virological response was more frequent in HCV genotype 2; however, hematologic response did not correlate with the viral genotype. One of the greatest accomplishment of this study was the demonstration of the efficacy of AT in a wide range of HCV-positive low-grade NHL subtypes other than splenic marginal zone lymphoma, as complete responses were actually observed without significant differences in all indolent NHL histologies. 

### 6.2. Recent Studies and Future Perspectives

Recently Mazzaro et al. [75] reported a comparison of PEG-interferon and standard interferon (plus ribavirin) as first-line treatment in 18 patients with HCV-positive low-grade B-cell NHL (1 follicular lymphoma, 1 splenic lymphoma with villous lymphocytes, and 16 lymphoplasmacytic lymphomas). Complete responses as well as SVR were higher in the group treated with PEG-interferon (6/10 patients, 60%) with respect to the group treated with standard interferon (3/8 patients, 37%). Achievement of hematological response significantly related to the disappearance of HCV-RNA, as all patients who experienced SVR developed hematological CR. In the previously cited study on subcutaneous “lipoma-like” extranodal marginal zone B-cell lymphoma of MALT [78], one patient was treated with interferon and ribavirin as first- line treatment and obtained a rapid virological response and a partial lymphoma response. Interestingly, another patient who relapsed 20 months after CHOP therapy plus radiotherapy was treated with interferon alone for 6 months and achieved a complete regression of nodules after 1 month of therapy together with virological response. Eight months after stopping AT HCV-RNA returned positive and 3 months later a subcutaneous lymphoma relapse occurred.

 In perspective, several lines of future clinical research can be pursued with the aim to further improve these results. First, it has to be investigated if the combination of rituximab and AT tested in symptomatic cryoglobulinemia by Saadoun and colleagues (rituximab weekly for 4 doses followed by PEG-interferon weekly plus ribavirin daily for 48 weeks) [14] is able to obtain a better long-term control of disease also in indolent B-cell NHL. Second, it has to be tested if new antiviral combinations with new anti-HCV agents (i.e., PEG-interferon and ribavirin plus boceprevir or telaprevir), that guarantee better rates of SVR in genotypes 1 hepatitis, could allow to increase the rate of hematologic response also in patients with more resistant genotypes. Finally, it has to be explored if future interferon-free regimens with direct antiviral agents only, could consent the access to AT also for HCV-positive NHL patients with contraindications to interferon use, for example because of advanced age, cytopenias, and/or comorbidities.

### 6.3. Role of Antiviral Therapy in Aggressive B-Cell Lymphomas

As previously mentioned, AT seems to be less efficacious than standard immune-chemotherapy for HCV-positive aggressive lymphomas (diffuse large B-cell lymphoma and mantle cell lymphoma) with respect to indolent ones. This is probably related to the lost of antigen dependence resulting from acquisition of additional mutations that are possibly responsible of more aggressive behaviour, although anecdotal cases of diffuse large B-cell lymphoma [79] and mantle cell lymphoma [80] treated with AT and obtaining remission have been reported. Nevertheless, many researchers have explored the option to integrate AT in the context of immune-chemotherapy programs in HCV-positive diffuse large B-cell lymphomas. Although some rare cases of concurrent delivery of AT and immune-chemotherapy with the aim to prevent or to treat hepatitis flares have been reported [81], treatment with interferon or PEG-interferon with or without ribavirin is usually not feasible because of hematologic toxicity, as showed by a pilot study by Musto and coworkers in 4 patients with diffuse large B-cell lymphoma [82]. The same authors explored the option to perform sequential AT with PEG-interferon and ribavirin for 3 months in responding HCV-positive patients with diffuse large B-cell lymphomas after 6–8 cycles of R-CHOP. They found that this strategy was effective, better tolerated and resulted in high rate of virus clearance in the first 12 patients treated. These preliminary data have been indirectly supported by a subsequent study that described the outcome of 69 HCV-positive NHL patients treated with chemotherapy; of them, 25 (14 with aggressive NHL and 11 with indolent NHL) subsequently underwent to AT and 8 out of 25 achieved an SVR [83]. None of the HCV-positive patients who obtained a SVR experienced NHL recurrence, while 29% of the nonresponders relapsed. Moreover AT resulted associated with better disease-free survival in multivariate analyses. These preliminary experiences confirmed that AT is not feasible in concomitance with immune-chemotherapy in HCV-positive DLBCL because of an excess of hematologic toxicity. On the other hand they suggest that a course of AT in patients in remission after immune-chemotherapy appears to be an attractive option with the aim to prevent hepatitis reactivation in view of long-term control of the lymphoma. However these data have to be confirmed in larger series and preferentially in prospective manner.

## 7. Conclusions

 In conclusion, anti-HCV treatment with interferon and ribavirin seems to be indicated for indolent B-cell NHL subtypes (in particular of marginal zone type) that not need immediately conventional immunochemotherapy. The lymphoma regression observed in some patients with interferon-based treatment is strongly in favour of a causative role of HCV in a subset of patients with indolent NHL. For this reason, it is likely to foresee that future improvements in AT may directly result in increase in cure rates of HCV-associated indolent NHL.

## Figures and Tables

**Table 1:** Clinical pathological studies describing indolent NHL subtypes associated with HCV infection.

	Year	Diagnosis	N^°^ pts	N^°^ pts tested for HCV	*N* HCV+ (%)	Genotypes	Cryoglobulinemia, *N* (%)
Arcaini et al. [16]	2006	SMZL	309	255	49 (19)	1b (*n* = 10), 2b (*n* = 1), 2a/2c (*n* = 4)	13 (10)
Saadoun et al. [17]	2005	SLVL	18	18	18 (100)	1 (*n* = 7), 2 (*n* = 4), 3 (*n* = 1), 4 (*n* = 1)	18 (100)
Arcaini et al. [18]	2007	NMZL	47	38	9 (24)	NA	2 (14)
Arcaini et al. [19]	2006	MALT-MZL Skin Salivary glands Orbit Other sites	172 *29 * *32 * *25 * *66 *	172 *29 * *32 * *25 * *66 *	60 (35%) *21 (43) * *15 (47) * *9 (36) * *15 (22) *	1a (*n* = 11), 1b (*n* = 1), 2a/2c (*n* = 10)	NA
Ferreri et al. [20]	2006	MALT-MZL orbit	55	55	7 (13)	NA	2 (29)
Paulli et al. [21]	2009	Subcutaneous MALT- MZL	13	13	13 (100)	2a/2c (*n* = 4), 2a (*n* = 2) 2b, (*n* = 1)	3 (75)
Tedeschi et al. [22]	2009	WM	140	140	21 (15)	NA	10 (48)
Arcaini et al. [23]	2011	WM SMZL	122 98	66 92	6 (9) 25 (27)	NA NA	0 3
Goldaniga et al. [24]	2008	B-CLPD	156	113	6 (5)	NA	NA

SMZL: splenic marginal zone lymphoma; NMZL: nodal marginal zone lymphoma; SLVL: splenic lymphoma with villous lymphocytes; MZL: marginal zone lymphoma; FL: follicular lymphoma; LPL: lymphoplasmacytic lymphoma; MCL: mantle cell lymphoma; SLL: small lymphocytic lymphoma; NHL: non-Hodgkin's lymphoma; B-CLPD: B-cell chronic lymphoproliferative disorders.

**Table 2:** Antiviral treatment in patients with indolent B-cell lymphoma associated with HCV infection.

	Year	N^°^ pts	Diagnosis	Genotypes	Cryoglobulinemia	Antiviral treatment	Virologic response	NHL response
Bauduer [63]	1996	1	MZL of MALT (oral cavity)	NA	—	*α*-IFN	1	1 PR
Caramaschi et al. [64]	1999	1	MZL of MALT (salivary glands)	NA	—	*α*-IFN	NA	1 CR
Moccia et al. [65]	1999	3	SMZL	NA	—	*α*-IFN	NA	2 CR
Patriarca et al. [66]	2001	1	LPL	2a/2c	—	*α*-IFN	1	1 CR
Hermine et al. [5]	2002	9	SLVL	NA	6	*α*-IFN	7	7 CR
Casato et al. [67]	2002	1	Leukemic MZL	NA	1	*α*-IFN	Decreased HCV-RNA	1 CR
Pitini et al. [68]	2004	2	SMZL	NA	—	*α*-IFN	2	2 CR
Tursi et al. [69]	2004	16	MZL of MALT (stomach)	NA	—	*α*-IFN-2b + RBV	11/16	16 CR
Kelaidi et al. [70]	2004	8	SMZL (*n* = 4) Disseminated MZL (*n* = 1) Leukemic MZL (*n* = 1) MZL of MALT (*n* = 2) (1 duodenus; 1 ileus)	3a (*n* = 1), 5a (*n* = 1) — 1b 4c/4d	8	*α*-IFN-2b + RBV	5 SVR, 2 PR	5 CR
Vallisa et al. [71]	2005	13	SMZL (*n* = 4) NMZL (*n* = 2) MZL of MALT (*n* = 2) FL (*n* = 1) LPL (*n* = 4)	1b (*n* = 2), 2b (*n* = 1) 2a/2c (*n* = 1), 1b (*n* = 1) 1b (*n* = 2) 2a 2a/2c (*n* = 1), 1n (*n* = 1), na (*n* = 2)	5	Peg-IFN + RBV	7 SVR, 1 PR	7 CR, 2 PR
Svoboda et al. [72]	2005	1	MZL of MALT (salivary gland, liver)	2b	—	Peg-IFN + RBV	1	CR
Saadoun et al. [17]	2005	18	SLVL	1 (*n* = 7) 2 (*n* = 4) 3 (*n* = 1) 4 (*n* = 1)	18	*α*-IFN (+ RBV in 10)	14 CR, 4 PR	14 CR, 4 PR
Paulli et al. [21]	2009	2	Subcutaneous MZL of MALT	2a/2c 2b	2	Peg-IFN + RBV	2 CR	1 CR, 1 PR
Oda et al. [73]	2010	1	B-NHL (liver)	2a	—	Peg-IFN + RBV	SVR	CR
Mauro et al. [74]	2012	1	LPL	1b	1	Peg-IFN + RBV (2nd line)	SVR	CR
Mazzaro et al. [75]	2009	18	1 SLVL 1 FL 16 LPL	1b (*n* = 11) 2a/2c (*n* = 7)	13	*α*-IFN + RBV (*n* = 8) Peg-IFN + RBV (*n* = 10)	9 SVR	9 CR, 4 PR

SMZL: splenic marginal zone lymphoma; NMZL: nodal marginal zone lymphoma; SLVL: splenic lymphoma with villous lymphocytes; MZL: marginal zone lymphoma; FL: follicular lymphoma; LPL: lymphoplasmacytic lymphoma; MCL: mantle cell lymphoma; SLL: small lymphocytic lymphoma; NHL: non-Hodgkin's lymphoma; NOS: not otherwise specified; IFN: interferon; RBV: ribavirin; CR: complete response; PR: partial response; SVR: sustained virologic response.
